# Risk perceptions of Italian paediatricians for the impact of climate change on children’s health

**DOI:** 10.1186/s13052-024-01736-4

**Published:** 2024-09-10

**Authors:** Sara Moraca, Luciana Indinnimeo, Paola De Nuntiis

**Affiliations:** 1 IRCCS Materno Infantile Burlo Garofalo, Trieste, Italy; 2 SIP- Società Italiana Di Pediatria, Rome, Italy; 3 Istituto Delle Science Dell’Atmosfera E del Clima- CNR ISAC, Bologna, Italy

**Keywords:** Climate change, Environment, Risk perception, Global health

## Abstract

**Backgrounds:**

This study delves into the risk perceptions of Italian pediatricians concerning climate change's impact on children's health. Given children's heightened vulnerability to climate-related health risks, comprehending these perceptions is crucial. A review of pertinent literature establishes the framework, emphasizing six key factors influencing children's susceptibility to climate-related health hazards.

**Methods:**

Methodologically, the study utilized a survey tool developed collaboratively with the Italian Society of Pediatrics (SIP), garnering responses from a representative sample of Italian pediatricians.

**Results:**

Findings indicate a high level of awareness among respondents regarding climate change and its health implications, with a majority attributing it primarily to human activity. Pediatricians recognize various current and anticipated health impacts of climate change, notably concerning illnesses linked to outdoor air quality. Despite acknowledging their role in addressing climate-related health concerns, respondents also cite barriers to engagement, including time constraints and knowledge gaps. However, they express interest in resources like professional training and policy statements to bolster their capacity for effective communication and advocacy.

**Conclusions:**

Comparisons with prior studies highlight the consistency of findings across diverse contexts and underscore the significance of integrating climate and environmental health education into medical training. Overall, this study sheds light on pediatricians' perspectives in tackling the convergence of climate change and children's health, pinpointing avenues for enhancing their involvement in climate advocacy and mitigation efforts.

## Introduction

Environmental and dietary exposures affect children's health from before conception (e.g. parental diet) and continue throughout pregnancy, infancy, and adolescence [14]. Many factors influence the susceptibility to or accentuate the effects of these exposures in children in different ways than they do in adults [1, 5, 15]. Six climate change-related factors that affect children’s health are:

- Physiological differences: Children are less able to adapt to heat than adults [1, 5, 15],
- Windows of vulnerability: Rapid organ development creates windows of vulnerability in utero and in early childhood. Prenatal or childhood experience of dysfunctions or exposure to infectious agents may be the source of childhood problems that can continue into adulthood [1, 5, 15],
- Increased exposure per unit of body weight: Babies eat, drink, and breathe more per unit of body weight than adults do, increasing their relative exposure to pollutants of all kinds when compared to adults [1, 5, 15],
- Different diets and behaviors: Children typically spend more time outdoors and eat more fruit and vegetables than adults, increasing their exposure to climate change [1, 5, 15],
- Increased longevity: Children typically have higher life expectancies than the generations that preceded them, potentially exposing them to new risks in the future [1, 5, 15],
- Dependence on adults: Children do not usually think about or manage their own health. Children’s health is typically the responsibility of the adults in their lives and, in a more general sense, the responsibility of those who make political and economic decisions that could affect their futures [1, 5, 15].

Approximately one of every five deaths worldwide occurs in a child under five years old. Lower respiratory tract infections, diarrhea, and malaria account for more than 50% of deaths in children under five, and all three could worsen with climate change [18]. Diarrhea is primarily attributable to environmental factors such as contamination of food and drinking water, conditions expected to become more frequent as temperatures and rainfall patterns change [18]. More than 35% of child mortality is due to malnutrition, and that is expected to rise as climate change increases food insecurity. Micronutrient deficiency may also exacerbate infection-related morbidity [18].

The WHO estimates that more than 88% of climate change-related diseases affect children five and under in high-income as well as in low- and middle-income countries (Zhang et al. 2017). However, the disease burden is not evenly distributed: in high-income countries this percentage drops to five percent, and in middle-income countries it is just 31% [20], while it is much higher in low-income countries. Globally, for all ages, it is estimated that 150,000 people died from diseases attributable to climate change in 2000 (0.3% of total deaths that year), accounting for 5.5 million Disability Adjusted Life Years (DALYs) lost (0.4% of total loss). However, these estimates only include deaths caused by excess temperatures, diarrhea, malnutrition, flood-related diseases, or malaria [18]. Climate change will probably increase the vector-, food- and waterborne infectious disease burden, especially malaria, dengue fever, and tick-borne diseases like Lyme disease [1, 5, 16]. Complications from these diseases are frequent in children, particularly for malaria [1, 5, 16].

Climate change is likely to worsen malnutrition because of its impact on agriculture, but the impact is difficult to quantify because it is the result of composite macrosocial factors [1, 5, 15]. However, the impact of climate change on agriculture will likely be the largest driver of climate change-related morbidity and mortality [15, 19].

Climate change can potentially change exposure to many pollutants, including exposure to ozone in some regions, because elevated temperatures can affect chemical reaction rates and pollutant transport mechanisms [1, 5, 15]. Changing rainfall patterns and temperatures also affect the pollen season, with changes in the severity and frequency of asthmatic and allergic events [1, 5, 15].

The frequency and severity of extreme weather events will impact child health by increasing food security, chronic mental illness, acute physical illness, water and food contamination, and forced migration that may exacerbate health problems, economic development, political instability, poverty, and civil unrest, further contributing substantially to the global disease burden [18]. Extreme weather events may also adversely affect pregnancy outcomes [3], as well as cause acute respiratory and renal damage along with potential effects on cognitive performance [1, 5]. An estimated 66.5 million children worldwide were affected by climate, natural, and man-made disasters annually in the 1990s. Current estimates, however, expect 175 million children per year to be affected by disasters in the future [2].

## Materials and methods

After carrying out a literature review on the subject, we decided to use the tool proposed by Kotcher (2021) because it appeared to be the most suitable for the purpose of this investigation. The study was conducted with the collaboration of SIP, the Italian Society of Pediatrics. That is the main scientific society of italian pediatricians and it was born back in 1898. It counts around 10.521 members involving university, hospital, family and community pediatricians. To understand how SIP members can be a representative sample of the Italian pediatrician population, we compared data from the Italian Ministry of Health and Eurostat data. Using instead Eurostat data, updated to 2020 (i.e. the newest available), there would be 16559 pediatricians in Italy.

The data refers to “general pediatricians”, therefore not distinguishing between public and private pediatricians, as occurs in the database of the Ministry of Health. For the database of Ministry of Health There are 7,022 public pediatricians.

The approval and distribution steps of the survey were as follows:

- sending the Italian translation of the original instrument to the SIP board of directors;
- adaptation of the tool according to the requests of the SIP; only one final question has changed, as already explained above;
- the tool, adapted, has been uploaded to Lime Survey. A link has been created, which can then be sent by email to potential respondents;
- test of the survey on 15 members of the SIP board of directors to verify that the tool was clear and exhaustive;
- sending of the survey to the entire SIP address book, via a newsletter created ad hoc and which was sent every Wednesday by the SIP secretariat.

The survey was active from 23 December 2021 to 31 March 2022.

A total of 1510 responses were collected, with a total rate response of 14,15%.

The survey took approximately 10 min to complete, was available only for SIP members and consisted of close-ended questions or multiple-choice answers. In particular, the questionnaire was divided into four sections: general climate change beliefs and attitudes, risk perceptions, communication barriers and resources, perceived role of health professionals.

Put states about: they were free to skip one or more answers (why is it better? find in the literature), with the consequence that some questions have more answers than others. The number of respondents out of the total number of participants is present in the raw data.

## Results

80% of the pediatricians who answered the questionnaire think that climate change is happening, while only 1% believe it is not happening and 18% preferred not to answer the question by selecting the “I don't know/I don't answer” option. 79% of respondents believe that climate change is caused solely or mainly by human activity, 18% believe it is caused equally by natural changes and human action, 3% believe it is caused by natural changes.

96% of respondents are somewhat concerned about climate change, 4% say they are not very worried and only 1%.

Respondents assessed the consensus among scientists regarding the anthropogenic origins of climate change as follows: approximately 1% of respondents think it ranges from 0 to 30%, 17% of respondents believe it is between 40 and 60%, 42% believe the range is between 70 and 80%, 40% believe it is between 90 and 100%.

### Risk perceptions of climate change as a human health threat

For over 90% of respondents, climate change will harm them personally, their community, people in their country and future generations of people.

Pediatricians recognize that climate change has several health effects already in the present.

The effect to which paediatricians attribute the greatest importance are the illnesses due to reduced outdoor air quality, with a figure much higher than the average of the other items: considering the answer options “a great deal” and “somewhat”, the percentage of respondents is 92%.

**Fig. 1:**
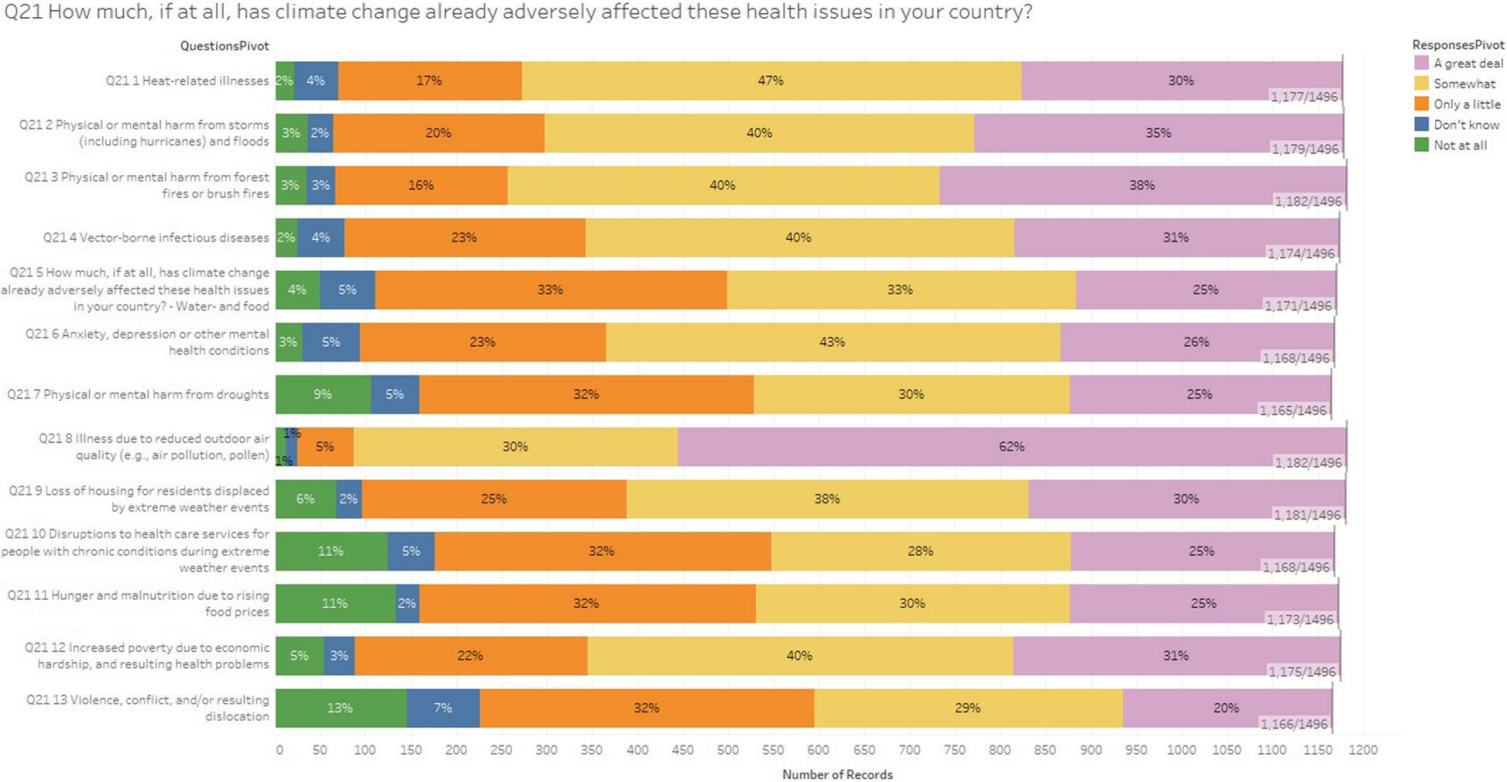
The survey question, “How much, if at all, has climate change already adversely affected these health issues in your country?” This bar chart illustrates the public perception of how climate change has adversely affected various health issues in their country. Respondents were asked to rate the impact of climate change on specific health problems, such as heat-related illnesses, physical or mental harm from storms, floods, forest fires, and brush fires, vector-borne infectious diseases, and water- and food-related issues. Additionally, the survey considered mental health conditions like anxiety and depression, harm from droughts, illnesses due to reduced outdoor air quality, and the loss of housing due to extreme weather events. Disruptions to healthcare services for chronic conditions during extreme weather events, hunger and malnutrition due to rising food prices, increased poverty due to economic hardship, and resulting health problems were also included, along with violence, conflict, and resulting dislocation. The chart categorizes responses into five groups: “Not at all,” “Don't know,” “Only a little,” “Somewhat,” and “A great deal.” For most health issues, a significant portion of respondents indicated that climate change has impacted these areas either “Somewhat” or “A great deal.” Notably, 62% reported that illnesses due to reduced outdoor air quality have been affected “A great deal,” while 47% said heat-related illnesses have been affected “Somewhat.” Other health concerns, like mental health conditions and harm from droughts, also show considerable perceived impact, with high percentages indicating these issues are affected “Somewhat” or “A great deal.” Overall, the chart highlights widespread concern about the negative effects of climate change on health, demonstrating that many people believe these issues are already being significantly affected

Between 70 and 78% (Fig. 1) believe that the most significant health impacts of climate change will be related to heat related illness (77%), physical or mental harm from storms and floods (75%), physical or mental harms from forest fires or bush fires (78%), vector borne diseases (71%), increased poverty due to economic hardship and resulting health problems (71%).

Between 49 and 69% of respondents stated that climate change is already having an impact on water and food born diseases (58%), physical or mental harm from drough (55%), loss of housing for displaced residents by extreme weather events (68%), disruptions to health care services for people with chronic conditions during extreme weather events (53%), hunger and malnutrition due to increased food prices (55%), violence, conflict and/or resulting dislocations ( 49%).

**Fig. 2:**
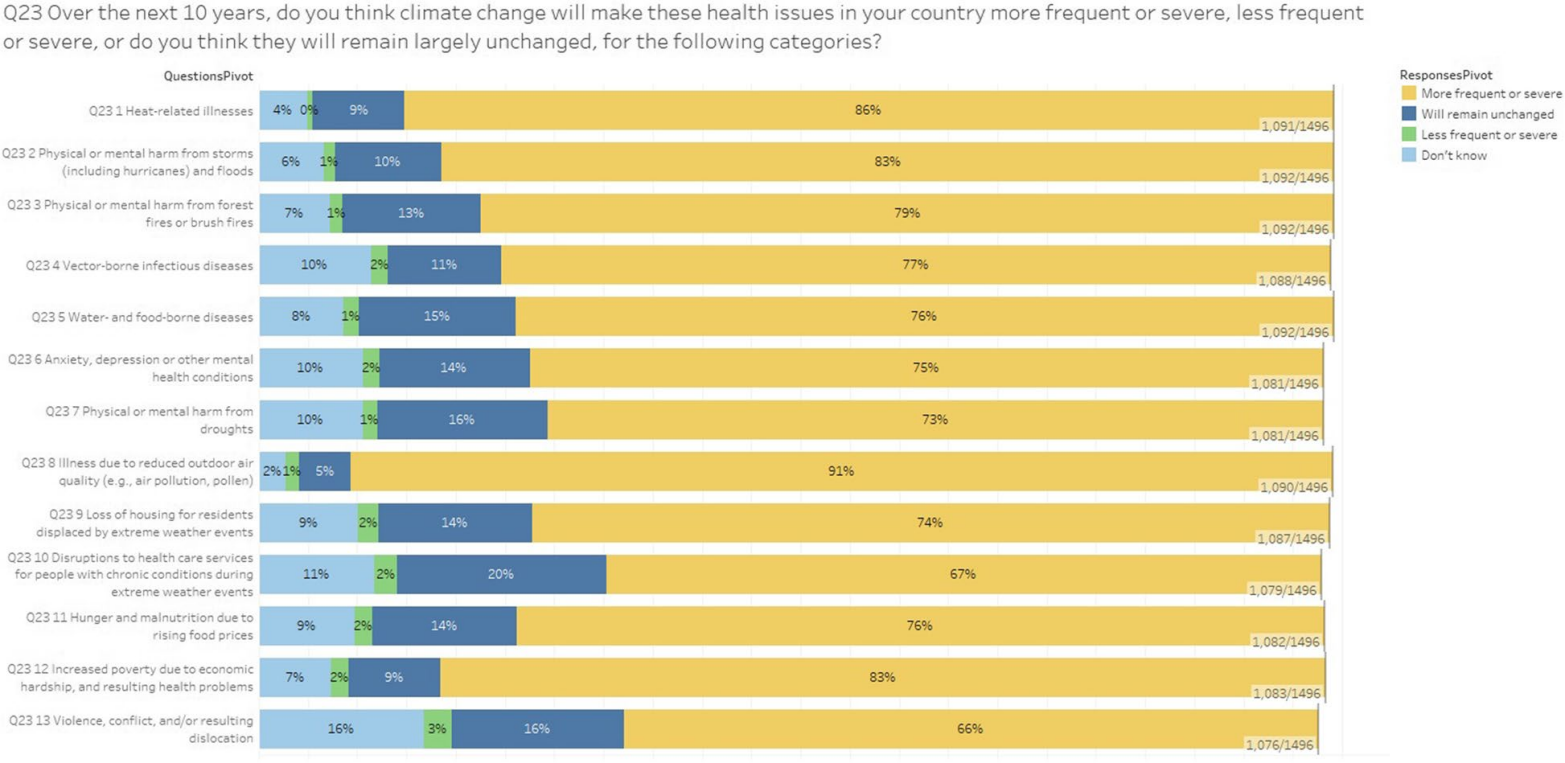
The survey question, “Over the 10 next years, if at all, do you think climate change make these health issues in your contry more frequent or severe, or do you think they will remainlargely unchanged, for the following categories”? This bar chart presents public opinions on how climate change will impact various health issues over the next 10 years. Respondents were asked whether they think these health issues will become more frequent or severe, less frequent or severe, or remain largely unchanged. The health issues considered include heat-related illnesses, physical or mental harm from storms (including hurricanes) and floods, forest fires or brush fires, vector-borne infectious diseases, water- and food-borne diseases, anxiety, depression, or other mental health conditions, harm from droughts, illness due to reduced outdoor air quality, and loss of housing for residents displaced by extreme weather events. Additional categories include disruptions to healthcare services for people with chronic conditions during extreme weather events, hunger and malnutrition due to rising food prices, increased poverty due to economic hardship, and resulting health problems, and violence, conflict, and/or resulting dislocation. The chart categorizes responses into four groups: “More frequent or severe,” “Will remain unchanged,” “Less frequent or severe,” and “Don't know.” A significant majority of respondents believe that most of these health issues will become “More frequent or severe” due to climate change. For example, 91% expect illnesses due to reduced outdoor air quality to become more frequent or severe, while 86% believe the same for heat-related illnesses. Similarly, 83% of respondents anticipate that physical or mental harm from storms and floods will increase in frequency or severity. Other notable findings include 79% of respondents expecting more frequent or severe harm from forest fires, 77% for vector-borne infectious diseases, 76% for water- and food-borne diseases, and 75% for anxiety, depression, or other mental health conditions. The perception of increasing frequency or severity extends to physical or mental harm from droughts (73%), loss of housing due to extreme weather events (74%), disruptions to healthcare services (67%), and increased poverty and related health problems (83%). Overall, the chart reveals a widespread concern that climate change will exacerbate a wide range of health issues in the coming decade

Between 67 and 91% of respondents think these impacts will become more severe and frequent in the next future (Fig. 2), between 5 and 20% believe they will remain the same, between 1 and 3% think the impacts will be less severe and frequent.

### The engagement with the public and the policy makers and the role of professional societies

Nearly all respondents (92%) acknowledge the responsibility that pediatricians have in bringing the health effects of the climate crisis to public attention, both as individual professionals and as a group. A slightly higher percentage of respondents (95%) also recognizes this responsibility towards policy makers.

The same amount of respondents think that pediatricians should actively encourage their national institutions to achieve the Paris Agreement goal on climate change mitigation.

94% of respondents think that pediatricians should actively encourage institutions at international level to achieve climate goals.

Despite this, only 36% of respondents would certainly agree to participate in a campaign to urge international institutions to act on climate change. 25% say they would support a campaign, but would not participate personally and 38% would perhaps participate but would like more information.

86% of respondents think that professional societies should provide the opportunity for members to participate virtually in meetings to cut emissions; for 70% of the respondents it should cut any ties that it may have with fossil fuel companies, including divestments, while 24% of the respondents believe that this is a substantially indifferent element.

### Barriers to engagement and helpful resources

Respondents acknowledge that there are some barriers to the willingness to communicate the effects of climate change on health to the public.

According to the respondents, the main barriers would be lack of time (54%) and lack of knowledge (52%).

39% of respondents believe that even if they do so, it would make no difference, 36% believe they are not supported by their peers and 38% believe the topic is too controversial. Finally, about a fifth of respondents (19%) believes that this activity it is too risky personally or professionally.

The majority of respondents clarified that some resources could be very useful or moderately useful for communicating the impact of climate change on health: policy statements by professional associations (91%), continuing professional education on climate change and health (97%), patient education materials (90%), guidance on how to make my workplace sustainable (88%), training to communicate effectively climate change and health (94%), action alert on when and how to advocate with policy makers (93%).

## Limitations

The results of the survey were collected during the pandemic period, this may have conditioned the answers given by pediatricians.

The pandemic could also have affected the participation of pediatricians, who in that historical period were subjected more than others to increasing work pressure.

In some of the questions the number of answers drops drastically, so they may be less representative than in others of the sample.

## Discussion

This is not the first survey on the perceptions of climate change conducted among a sample of Italian pediatricians. An earlier survey [9] was conducted in 2022 among a smaller sample (117 respondents) who were members of the Italian Pediatric Respiratory Society (SIMRI). The earlier survey contained more specific questions, mostly relating to the impact of climate change on children's lung and respiratory health.

While participants in the earlier survey were primarily university pediatricians, our sample is composed of family pediatricians, hospital pediatricians, training pediatricians, and includes only a small number of university pediatricians.

The literature on the perception of health professionals about the impacts of climate change on human health is growing, and several studies [4, 8] have found that most health professionals have at least a basic knowledge about climate change, and some are already perceiving its effects on human health, though there is wide variation among health professionals. However, to best contextualize our study, we are confining our focus to reviewing here those few studies that have considered both pediatricians. In the literature, we found only two studies that center the perceptions of pediatricians on the effects of climate change on health: the first is the study of a sample of Italian pediatric pulmonologists mentioned above, and the other is a pilot study [6] conducted among 66 Academic Pediatric Association members with a stated interest in environmental health.

The percentage of pediatricians in our study who acknowledge the existence of climate change, recognize its effects on health, and are interested in learning more about the topic is in line with previous studies. In the pilot study, 94% of the pediatricians agreed that “Health professionals have a responsibility to understand the impact of climate change on human health”. In the study of Italian pediatric pulmonologists, 76% of respondents totally agreed with the *Lancet* statement “Climate change represents the greatest global health threat of the twenty-first century”, with 22% partially agreeing. In our study, 80% of respondents said they believed that climate change is happening, while 18% of respondents preferred not to comment on the matter. 18% of respondents also believe that climate change is the result of both natural and human causes equally.

We found three other similar pediatrician surveys [11–13], two of which employed a tool very similar to the one used for our survey (the group that developed our tool is the group that conducted those three studies). Unfortunately, however, the responses of the pediatricians are not identifiable and separable in any of these three studies.

Two of the surveys were conducted by the American Thoracic Society (ATS), one in the US and one internationally, while the third was conducted by the American Academy of Allergy, Asthma & Immunology (AAAAI).

These three surveys show that a similar percentage of respondents (96%, 89%, and 81%) think that climate change is happening and that it is mostly or solely determined by human action (70%,70%, 54%).

The survey conducted among SIMRI members shows that, as in our study, pediatricians recognize the impacts of air quality on children's health, but we can’t make a more direct comparison given the differences in questions. However, the result is particularly important given that the impact of climate change on air quality, among the items available in our questionnaire, is the one that was most often selected by pediatricians.

82% of SIMRI survey respondents also stated that pollen-induced rhinitis and asthma have worsened, and approximately 77% of respondents believe the pollen production window has changed (starting earlier or starting earlier and finishing later). Similar results emerge from the survey conducted among AAAI members. After respondents rated a series of items related to climate change and air quality, they were asked to clarify which items had a current impact on health and which they thought would have an impact in the next 10–20 years. 73% of respondents thought air pollution would increase the severity of chronic diseases like asthma, COPD, pneumonia, and cardiovascular disease; 63% thought visits to doctors’ offices or Emergency Departments would increase in response to allergic sensitization and exposure to plants or mold; and 49% thought injuries due to severe storms, droughts, and fires would increase.

In the two ATS surveys, ATS members were asked about the impact of a series of items on current and future health. These items, similar to those included in our survey, included injuries due to severe storms, floods, droughts, fires (2015: 57% and 69%; 2016: 69% and 77%); increased care for allergic sensitization & symptoms of plant/mold exposure (2015: 58% and 66%; 72% and 76%); heat-related effects (2015: 48% and 67%; 70% and 79%); vector borne infections (2015: 40% and 62%; 59% and 68%); air pollution related increases in severity of illness (2015:77% and 80%; 2016:88% and 89%); diarrhea from food/waterborne illnesses (2015: 26% and 48%; 2016: 55% and 65%). These responses show general agreement among health professionals who responded to those surveys.

When faced with identical or comparable items, participants in the ATS and AAAAI surveys responded in line with participants in our study, especially regarding future awareness, with most health professionals recognizing the frequency and severity of climate-related health impacts. Similar to our findings, the ATS surveys and the AAAAI survey found that respondents feel that engagement with the public and with policy makers is important, and, in fact, they think that health professionals have a role in informing the public about the health impacts of climate (ATS 2015: 72%; ATS 2016: 79%) (AAAAI: 56%). The role of advocacy and influencing policy makers is also recognized in the ATS surveys (2015: 75%; 2016: 85%) and in the AAAAI survey (65%). The APA pilot study conducted among 83 pediatricians found that three quarters of respondents (76%) said they took candidates’ stance on climate policies into account when voting, about half of respondents (49%) had contacted a legislator to convince them to take action on climate, a third (33%) declared they participate in making their institution environmentally sustainable, 29% declared that they have participated in a march, and about a fifth of respondents (about 20%) contacted one or more companies to ask them to be more sustainable, advocated at their local level, or lobbied for specific local changes.

Our survey findings were in line with the ATS and AAAAI surveys when it came to physicians’ willingness to communicate about climate change and health, their lack of time to communicate this information, their lack of knowledge on the topic, their low sense of self-efficacy on the topic, their lack of support from peers, and how discussing such a controversial topic can be risky on a personal or professional level.

Our data are in line with the ATS and AAAAI surveys in identifying resources for addressing gaps in knowledge and lack of support. These resources include policy statements by professional associations, continuing professional education on climate change and health, patient education materials, guidance on how to make workplaces environmentally sustainable, training for effective climate change and health communication, action alerts for when and how to advocate with policy makers. Most respondents (97%) to the SIMRI survey were interested in training to learn more about climate change-related health issues. However, despite a shared sense of responsibility to understand the health impacts of climate change, only 60% of the respondents to the Academic Pediatric Association's Special Interest Groups in Complementary and Integrative Medicine and Environmental Health Survey had attended a formal educational session about the health effects of climate change.

Numerous scientific societies and institutions [7, 10, 17] have recognized the need to integrate environmental and climate medicine into medical training, and there are numerous ongoing pilot initiatives to evaluate different ways of integrating these new curricula.

## Conclusions

As a first factor, while this study adds to the growing body of literature on the perceptions of health professionals regarding climate change, it specifically focuses on pediatricians' perspectives, thereby providing valuable insights into a crucial demographic given children's unique vulnerabilities.

Comparisons with previous surveys among Italian pediatricians and international health professionals reveal a consistent pattern of acknowledgment regarding climate change's existence, its primarily human-driven nature, and its health impacts. This consistency underscores the importance of addressing climate change in healthcare contexts.

Thirdly, the identified barriers to engagement, such as time constraints and knowledge gaps, are echoed across various studies, indicating a common challenge faced by healthcare professionals in effectively addressing climate-related health concerns.

Moreover, the willingness of pediatricians to receive training and access resources highlights opportunities for intervention, including the provision of professional development opportunities and the development of policy statements by professional associations.

Finally, the alignment of our findings with other surveys underscores the importance of collaborative efforts across scientific societies and institutions to integrate environmental and climate medicine into medical training curricula effectively.

## Data Availability

The datasets used and/or analysed during the current study are available from the corresponding author on reasonable request.
