# Correction: Risk perceptions of Italian paediatricians for the impact of climate change on children’s health

**DOI:** 10.1186/s13052-024-01787-7

**Published:** 2024-10-10

**Authors:** Sara Moraca, Luciana Indinnimeo, Paola De Nuntiis

**Affiliations:** 1 IRCCS Materno Infantile Burlo Garofolo, Trieste, Italy; 2 SIP- Società Italiana Di Pediatria, Rome, Italy; 3 Istituto Delle Science Dell’Atmosfera E del Clima- CNR ISAC, Bologna, Italy

**Correction to: Italian Journal of Pediatrics (2024) 50:170**

10.1186/s13052-024-01736-4

The original article erroneously presents a minor typo in Affiliation #1; ‘Garofalo’ should instead read ‘Garofolo’.

